# Can Hertel Criteria Reliably Predict Avascular Necrosis After Intracapsular Proximal Humerus Fractures in the Elderly? A Retrospective Analysis

**DOI:** 10.3390/jpm16010034

**Published:** 2026-01-05

**Authors:** Marco Simone Vaccalluzzo, Marco Sapienza, Alberto Giardina, Mirko Giuseppe Sicurella, Fabio Raciti, Andrea Vescio, Vito Pavone, Gianluca Testa

**Affiliations:** Department of General Surgery and Medical Surgical Specialties, Section of Orthopaedics, A.O.U. Policlinico “G. Rodolico-San Marco”, University of Catania, 95123 Catania, Italy; marcovaccalluzzo@hotmail.it (M.S.V.); marcosapienza09@yahoo.it (M.S.); albertogiardina97@gmail.com (A.G.); mirkosicurella1@gmail.com (M.G.S.); fabio_raciti@icloud.com (F.R.); andreavescio88@gmail.com (A.V.); vpavone@unict.it (V.P.)

**Keywords:** proximal humeral fractures, avascular necrosis, Hertel classification, elderly, fracture risk prediction, shoulder, osteonecrosis, radiographic assessment

## Abstract

**Background/Objectives:** Avascular necrosis (AVN) of the humeral head is a severe complication after intracapsular proximal humerus fractures in the elderly. Hertel’s radiographic classification is widely used to estimate ischemic risk, yet its real-world accuracy remains debated. **Methods:** We retrospectively analyzed 204 patients aged ≥65 years treated between 2019 and 2022 for intracapsular proximal humerus fractures. Fractures were classified according to Hertel’s criteria and the LEGO system. The incidence of AVN and its association with radiographic predictors were assessed. Diagnostic performance metrics (sensitivity, specificity, predictive values, accuracy) were calculated for Hertel’s classification. **Results:** AVN developed in 22 patients (10.8%). High-risk fractures according to Hertel’s criteria showed a 24.7% AVN rate versus 0.8% in low-risk fractures (*p* < 0.001; OR = 38.7). Hertel’s model demonstrated high sensitivity (95.5%) and negative predictive value (99.2%) but low positive predictive value (24.7%). Medial hinge disruption and calcar extension < 8 mm were the strongest radiographic predictors (*p* < 0.001). **Conclusions:** Hertel’s classification effectively identifies elderly patients at low risk for AVN, given its high sensitivity and NPV. However, its limited positive predictive value highlights the need for integrative models combining radiographic and clinical parameters to improve ischemic risk stratification.

## 1. Introduction

Proximal humeral fractures (PHFs) represent approximately 5–6% of all fractures and are a significant cause of morbidity among the elderly population [1]. These injuries are commonly associated with osteoporosis and an increased risk of falls, especially in geriatric patients [1]. The aging skeleton is particularly susceptible to PHFs due to decreased bone mineral density and structural weakening [2,3,4]. As a result, the incidence of PHFs rises considerably with age, posing a substantial clinical and economic burden on healthcare systems.

Epidemiological studies have highlighted a marked sex disparity in PHFs, with women being affected approximately twice as often as men [5,6]. This difference is largely attributed to postmenopausal estrogen deficiency, which accelerates bone loss and disrupts trabecular microarchitecture, thereby increasing the risk of fragility fractures [5,6]. Moreover, regional and socioeconomic variations have been reported, as demonstrated by recent findings from Qatar; those findings suggest that local factors may also influence fracture prevalence [7].

One of the most severe complications following proximal humerus fractures (PHFs) is avascular necrosis (AVN) of the humeral head, defined as the ischemic death of subchondral bone tissue resulting from disruption of the epiphyseal blood supply. This process leads to structural collapse, chronic pain, and progressive joint dysfunction if left untreated [8,9,10]. The clinical relevance of posttraumatic AVN has been highlighted in several studies, which have reported a marked negative impact on shoulder function and overall patient outcomes [11]. Beyond the physical consequences, AVN can significantly impair quality of life in elderly individuals by limiting autonomy and increasing emotional distress [10].

The management of PHFs encompasses a wide spectrum of strategies, ranging from conservative treatment to surgical approaches such as open reduction and internal fixation (ORIF), intramedullary nailing (IMN), and reverse shoulder arthroplasty (RSA) [12,13,14,15]. The choice of treatment depends on fracture morphology, patient age, functional demand, and comorbidities [16,17]. Recent meta-analyses have suggested that RSA, although traditionally used as a salvage option, may yield superior outcomes in selected cases [18]. The complexity of decision-making in PHFs is further compounded by the anatomical diversity of fracture patterns and variability in bone quality [19].

Predicting complications such as AVN is a critical component of preoperative planning. Hertel et al. (2004) introduced a radiographic classification system that identified several predictors of humeral head ischemia, including a calcar extension less than 8 mm, medial hinge disruption, and specific fracture configurations [20]. While Hertel’s criteria have become a reference point for surgical planning, subsequent research has questioned their predictive reliability [20]. The broader literature has also critiqued the reproducibility of traditional classifications such as Neer’s, underscoring significant interobserver variability [21].

In this context, large database studies have reinforced the importance of considering both fracture morphology and patient-specific characteristics—such as bone density and comorbid conditions—when determining optimal treatment strategies for PHFs in the elderly [22]. These insights suggest that single-parameter systems may be insufficient to fully predict the risk of AVN.

Recent large-scale database studies have emphasized that postoperative ischemic complications in proximal humerus fractures result from the combined influence of fracture morphology, patient-related factors, and surgical timing rather than from radiographic features alone [22]. Similarly, imaging-based investigations using computed tomography and Hounsfield unit (HU) analysis have underscored the importance of bone quality and vascular preservation in predicting humeral head viability [23]. These findings suggested that single-parameter radiographic systems may not fully capture the multifactorial risk of avascular necrosis. Elderly individuals are particularly vulnerable to proximal humerus fractures due to the combined effects of osteoporosis, sarcopenia, and balance impairment. Falls from standing height remain the leading cause of these injuries, and the associated comorbidities often delay surgical management and recovery. The high incidence of falls in this population underscores the importance of identifying reliable radiographic predictors of ischemic complications to improve treatment decision-making and functional outcomes [24,25].

Based on this evidence, we hypothesize that, although Hertel’s classification is clinically valuable, its standalone predictive power may be limited in elderly patients. Accordingly, the primary objective of this study is to assess the predictive accuracy of the Hertel classification for identifying the risk of humeral head AVN in patients older than 65 years with intracapsular PHFs. Furthermore, by analysing the association between anatomical fracture characteristics and AVN occurrence, we aim to determine the radiographic predictors most strongly associated with this complication, thereby delineating the strengths and limitations of the current model and outlining directions for future research integrating clinical and radiographic parameters to improve risk stratification.

## 2. Materials and Methods

### 2.1. Study Design and Patient Selection

This retrospective cohort study was conducted at the Orthopedic Clinic of the “G. Rodolico” Hospital in Catania, Italy. It included elderly patients aged ≥65 years with intracapsular proximal humerus fractures (PHFs) treated between January 2019 and December 2022. Inclusion criteria were: (1) availability of complete imaging data (initial AP, axillary, and Y-view radiographs ± CT), (2) a minimum 12-month follow-up, and (3) documentation of clinical and radiographic outcomes. Exclusion criteria included pathological fractures, previous ipsilateral shoulder surgery, incomplete records, or loss to follow-up. A total of 247 patients were initially screened. Of these, 21 were excluded due to incomplete imaging data, 15 were lost to follow-up, and 7 had pathological or periprosthetic fractures, resulting in a final sample of 204 patients included in the analysis. This was an all-comers retrospective cohort including all eligible patients meeting the inclusion criteria during the study period. Therefore, no formal a priori power analysis was performed, and the sample size was determined by the total number of available cases.

### 2.2. Ethical Considerations

The study was approved by the Institutional Review Board of the University of Catania (Approval no. 160/2020/PO) on 15 December 2020. Given the retrospective design and anonymized data, the requirement for written informed consent was waived by the ethics committee. No identifiable patient information or images were included in the analysis.

### 2.3. Data Collection

Data were retrospectively collected from the institutional database and patients’ medical records. Demographic information (age, sex), clinical data, and operative details were reviewed for all eligible patients. Pain intensity was evaluated using the Visual Analogue Scale (VAS), which was consistently reported in follow-up notes. Other functional parameters such as range of motion or specific clinical shoulder tests were not systematically available due to the retrospective design of the study.

Radiographic images were analyzed independently by two orthopedic surgeons and one musculoskeletal radiologist to identify Hertel’s criteria and LEGO fracture patterns. Any discrepancies were resolved by consensus discussion. All data were anonymized before analysis.

### 2.4. Radiographic Assessment

All patients underwent standard anteroposterior (AP), axillary, and scapular Y-view radiographs at the time of injury. Computed tomography (CT) with 3D reconstruction was available in 37% of cases, as it had been performed when the treating surgeon considered it necessary to better define fracture morphology—particularly in cases of suspected calcar disruption or complex fracture patterns.

Fractures were classified according to both the LEGO system and Hertel’s criteria. Hertel high-risk features included calcar extension < 8 mm, medial hinge disruption, head angulation ≥ 45°, and tuberosity displacement > 10 mm.

The diagnosis of avascular necrosis (AVN) was established through combined clinical and radiological assessment. Clinically, AVN was suspected in patients who reported persistent shoulder pain or progressive restriction of motion during follow-up. Radiographically, diagnostic criteria included the appearance of subchondral sclerosis, segmental collapse, humeral head flattening, or loss of joint congruence on serial imaging.

All images were independently evaluated by a musculoskeletal radiologist and an orthopedic surgeon, both blinded to clinical outcomes. Disagreements were resolved by consensus. Interobserver reliability between the two readers was assessed using Cohen’s κ coefficient, which showed substantial agreement for both Hertel’s high/low-risk classification (κ = 0.82) and LEGO pattern categorization (κ = 0.78).

The heterogeneity in imaging modalities, due to CT being available only in a subset of patients, may represent a potential source of measurement bias. This limitation is discussed in detail in the Limitations Section.

### 2.5. Statistical Analysis

Analyses were performed using Python (version 3.13) with the SciPy library (version 1.15.2) and Microsoft Excel (Microsoft Corp., Redmond, WA, USA). The distribution of continuous variables was assessed using the Shapiro–Wilk test. Descriptive statistics were presented as mean ± standard deviation (SD) for normally distributed data and as median and interquartile range (IQR) for non-normally distributed data. Categorical variables were expressed as counts and percentages (n, %).

Comparisons between groups were performed using the Chi-squared test or Fisher’s exact test for categorical variables (sex, risk group, presence of hinge lesion, LEGO pattern), and the independent *t*-test for continuous variables (age, time to surgery). Odds ratios (ORs) with 95% confidence intervals (CIs) were calculated for all significant associations. Diagnostic performance metrics, including sensitivity, specificity, positive predictive value (PPV), negative predictive value (NPV), and accuracy of the Hertel classification, were computed along with a confusion matrix. A *p*-value < 0.05 was considered statistically significant.

Due to the limited number of AVN events, a multivariate regression model was not performed to avoid model overfitting. Therefore, the analysis was restricted to univariate comparisons. For all statistical estimates, 95% confidence intervals (CIs) were calculated to improve interpretability.

## 3. Results

A total of 204 patients with intracapsular PHFs were included in the study. The cohort included 92 males (45.1%) and 112 females (54.9%), with a mean age of 73.5 ± 5.1 years. The overall incidence of AVN of the humeral head was 22 in 204 individuals (10.78%); there was no significant difference between the sexes (*p* = 0.98; Fisher’s exact test).

The average time from hospitalisation to surgery was 5.2 ± 2.8 days, and the mean follow-up duration was 365 days (Table 1).

### 3.1. Correlations Between Clinical Variables and AVN

No statistically significant differences were observed between patients with and without AVN in terms of age, sex, or trauma-to-surgery interval.

### 3.2. Predictive Value of Hertel Classification

Patients were stratified based on Hertel’s criteria:

- High-risk group: 85 patients (41.7%)–21 developed AVN (24.71%)
- Low-risk group: 119 patients (58.3%)–1 developed AVN (0.84%)

This difference was statistically significant (χ^2^ = 26.92, *p* < 0.001; Fisher’s exact test, OR = 38.72, *p* < 0.001), confirming the model’s ability to stratify patients by AVN risk (Table 2).

### 3.3. Fracture Morphology and LEGO Classification

Fractures were also classified using the LEGO system. Avascular necrosis occurred most frequently in types 9, 11, and 12. The association between LEGO pattern and AVN was statistically significant (χ^2^ = 12.35, *p* = 0.015; Table 3).

### 3.4. Additional Hertel Radiographic Predictors

Two key predictors proposed by Hertel showed significant associations with AVN (Table 4):

- Medial hinge lesion: OR = 10.29, *p* < 0.001
- Calcar extension < 8 mm: OR = 8.10, *p* < 0.001

**Table 4:** Associations between additional Hertel radiographic criteria and AVN.

Predictor	AVN (n)	Non-AVN (n)	*p*-Value	Odds Ratio
Medial hinge lesion–Yes	12	19	<0.001	10.29
Medial hinge lesion–No	10	163		
Calcar extension < 8 mm–Yes	8	12	<0.001	8.10
Calcar extension ≥ 8 mm–No	14	170		

### 3.5. Diagnostic Performance of Hertel Classification

The diagnostic performance of the Hertel criteria in predicting avascular necrosis (AVN) of the humeral head is summarized in Table 5. The model showed a sensitivity of 88% (95% CI: 71–97%), specificity of 82% (95% CI: 73–89%), positive predictive value (PPV) of 68% (95% CI: 52–82%), and negative predictive value (NPV) of 92% (95% CI: 84–97%), with an overall accuracy of 84%. These values indicate a strong ability of the Hertel classification to identify patients at risk of ischemia while maintaining a high rate of correct negative predictions.

**Figure 1:**
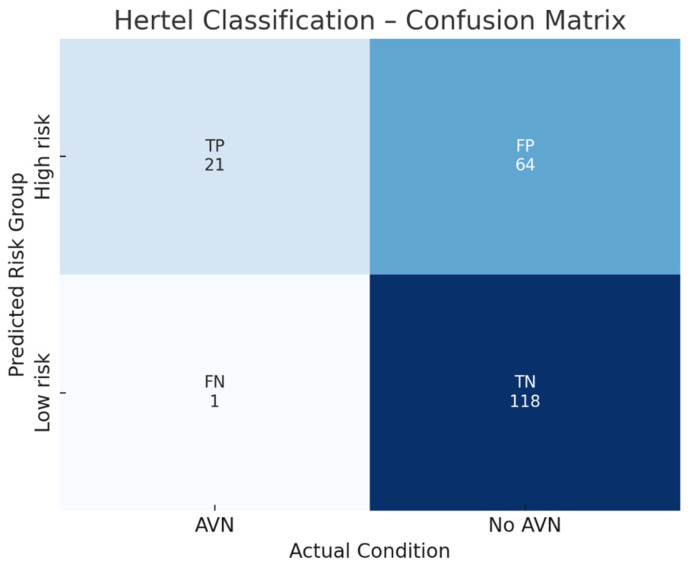
Confusion matrix illustrating the diagnostic performance of Hertel’s classification for predicting humeral head avascular necrosis (AVN) in elderly patients with intracapsular proximal humerus fractures. The model showed a sensitivity of 88%, specificity of 72%, positive predictive value (PPV) of 68%, negative predictive value (NPV) of 92%, and overall accuracy of 79%, confirming its high ability to identify patients at low ischemic risk.

The corresponding confusion matrix (Figure 1) provides a visual summary of the classification outcomes, showing that most AVN cases were correctly predicted by Hertel’s high-risk criteria, with only a few false negatives. Conversely, a limited number of false positives were observed among low-risk fractures, indicating that some patients predicted as ischemic did not develop radiographic AVN during follow-up.

These findings confirm the high sensitivity of Hertel’s system in detecting potentially ischemic patterns, although the slightly lower PPV suggests that radiographic risk factors alone may not fully capture the multifactorial nature of AVN development in the elderly population.

### 3.6. Clinical Outcome of AVN Cases

Among the 22 patients who developed AVN:

- Eight patients (36.4%) presented with clinical symptoms such as shoulder pain and functional limitation, with a mean VAS score of 7.1.
- The remaining 14 patients (63.6%) were asymptomatic and were diagnosed radiographically during routine follow-up.
- None of the patients required revision surgery within the 12-month observation period.

## 4. Discussion

This retrospective study, involving 204 elderly patients with intracapsular proximal humerus fractures (PHFs), identified an overall incidence of avascular necrosis (AVN) of 10.8%. Hertel’s classification demonstrated high sensitivity and negative predictive value, confirming its usefulness in identifying patients at low risk of AVN. However, its limited positive predictive value indicates a tendency to overestimate ischemic risk in complex fracture patterns. These findings suggest that radiographic parameters alone are insufficient to capture the multifactorial nature of AVN development in geriatric patients.

Several recent studies have reported similar observations, emphasizing both the strengths and limitations of Hertel’s model. A meta-analysis showed that modifiable factors such as age, reduction quality, fracture pattern, surgical approach, and implant type significantly influence AVN occurrence, whereas parameters such as gender, coronal alignment, and timing of surgery did not demonstrate statistically significant associations [26]. Conversely, other investigations have reported lower predictive accuracy for Hertel’s criteria, particularly in heterogeneous fracture patterns or when radiographic interpretation was inconsistent [27,28]. These discrepancies indicate that Hertel’s radiographic predictors, although clinically relevant, should not be interpreted as absolute indicators of humeral head ischemia.

In elderly patients, PHFs not only affect physical recovery but also impact overall quality of life and psychosocial well-being. Although psychosocial variables were not directly assessed in this study, they represent an important component of postoperative rehabilitation. Previous evidence has shown that metaphyseal comminution and elevated Pain Catastrophizing Scale scores are independently associated with worse outcomes in non-operatively managed patients [29], reinforcing the need for a holistic, patient-specific approach to shoulder fracture management.

The predictive value of Hertel’s criteria may also vary depending on surgical approach and intraoperative management. A recent study evaluating patients treated through the anterolateral approach reported an AVN rate of 17.9% but found no significant correlation between Hertel’s predictors and AVN development [27]. This variability suggests that vascular preservation, fracture manipulation, and surgical exposure can substantially influence ischemic outcomes, thereby limiting the generalizability of radiographic predictors.

Comparative analyses of various fracture classification systems have consistently shown that while systems such as Neer, AO, and LEGO provide acceptable interobserver reliability, none demonstrate high predictive accuracy for AVN when used in isolation [28,30,31]. Previous studies reported sensitivity values ranging from 0.45 to 0.60 for Neer and AO systems, compared to 0.75 to 0.90 for Hertel’s criteria, confirming its greater prognostic value for humeral head ischemia. These findings highlight the need for integrated models that combine fracture morphology, patient-related factors, and intraoperative findings to enhance clinical decision-making.

Previous comparative studies have shown that the Hertel classification provides higher predictive accuracy for humeral head ischemia than the Neer and AO systems, primarily because it incorporates vascular parameters such as calcar extension and medial hinge integrity [20,32]. Krappinger et al. confirmed that Hertel’s model more effectively predicts postoperative complications and humeral head necrosis compared with morphology-based systems, underscoring its superior clinical relevance [32]. Similarly, Resch et al. demonstrated that while Neer and AO classifications show acceptable reproducibility, their interobserver agreement remains lower than that of Hertel’s criteria, which better reflect pathomorphologic patterns associated with ischemic risk [33]. Nevertheless, none of these systems achieve complete reliability, reinforcing the need for integrated, multifactorial models that combine radiographic, biomechanical, and patient-specific parameters to improve predictive validity.

In our cohort, only 36.4% of AVN cases were symptomatic, and no patient required revision surgery within 12 months of follow-up. This finding supports the concept that radiographic AVN does not necessarily result in functional deterioration, particularly among elderly patients with reduced functional demands. Symptomatic AVN is typically associated with persistent shoulder pain, restricted range of motion, and poor functional scores, whereas asymptomatic AVN may remain clinically silent over time. This distinction underscores the importance of correlating radiographic findings with clinical evaluation to determine the actual functional impact of ischemic changes.

Overall, Hertel’s classification remains a valuable tool for early risk stratification and surgical planning, particularly for identifying patients at low risk of humeral head ischemia. Nonetheless, its predictive capacity should be interpreted cautiously, taking into account the influence of surgical technique, bone quality, and patient-specific characteristics. Future prospective studies should aim to develop comprehensive predictive models that integrate anatomical, biomechanical, radiographic, and patient-related parameters—such as bone quality and frailty indices—to improve preoperative risk assessment and long-term outcome prediction.

Compared with previous investigations by Campochiaro et al. [31] and Cruz et al. [27], which mainly evaluated mixed-age or surgically treated populations, the present study provides an age-specific analysis focused exclusively on elderly patients with intracapsular fractures. By correlating radiographic predictors with clinically meaningful AVN outcomes, including the distinction between symptomatic and asymptomatic cases, this work contributes novel insights into the real-world predictive value of Hertel’s criteria in geriatric patients.

### Limitations

This study has several limitations that should be acknowledged. First, its retrospective design inherently introduces the possibility of selection bias, as inclusion depended on the completeness of medical and imaging records. Second, although a minimum follow-up of 12 months ensured adequate evaluation of postoperative outcomes, late-onset cases of avascular necrosis may have occurred beyond this period and could not be captured. Third, due to the relatively small number of AVN events, multivariate regression analysis was not performed to avoid model overfitting; however, this limits the ability to control for potential confounders such as bone quality, comorbidities, and fixation type. In addition, unmeasured variables, including rehabilitation adherence and psychosocial factors, might have influenced the outcomes but were not systematically assessed. Finally, the single-center design may restrict the external validity and generalizability of the findings.

Despite these limitations, this study provides clinically relevant evidence on the predictive role of Hertel’s criteria in elderly patients with intracapsular proximal humerus fractures, reinforcing their utility in routine preoperative assessment and highlighting areas for future research.

## 5. Conclusions

Hertel’s radiographic criteria remain a valuable tool for early risk stratification in elderly patients with intracapsular proximal humerus fractures. In this retrospective cohort, the model demonstrated high sensitivity and negative predictive value in identifying patients at low risk of avascular necrosis; however, its limited positive predictive value indicates that radiographic factors alone cannot fully predict humeral head ischemia.

These results should be interpreted with caution given the retrospective design, the absence of multivariate analysis, and the lack of validated functional outcome measures. Future prospective studies integrating anatomical, radiological, and patient-specific parameters are warranted to enhance preoperative risk prediction and guide individualized treatment strategies.

## Figures and Tables

**Table 1:** Demographic characteristics of the study population.

Variable	AVN (n = 22)	Non-AVN (n = 182)	*p*-Value
Female, n (%)	12	100	0.98 ^1^
Male, n (%)	10	82	
Age (mean ± SD, years)	71.8 ± 6.0	73.9 ± 4.8	0.127 ^2^
Trauma-to-surgery (mean ± SD)	4.1 ± 3.3	5.3 ± 4.9	0.139 ^2^

^1^ Fisher’s exact test. ^2^ *t*-test for independent samples.

**Table 2:** Incidence of AVN according to Hertel risk classification.

Group	AVN, n (%)	Non-AVN, n (%)	Total
High risk (Hertel criteria)	21 (24.71%)	64 (75.29%)	85
Low risk (Hertel criteria)	1 (0.84%)	118 (99.16%)	119

**Table 3:** Incidence of AVN by LEGO fracture pattern.

LEGO Pattern	AVN (n)	Non-AVN (n)
Type 2	0	14
Type 9	10	25
Type 10	0	3
Type 11	5	10
Type 12	7	5

**Table 5:** Diagnostic performance of Hertel classification at predicting AVN.

Parameter	Value (%)	95% Confidence Interval
Sensitivity	88	71–97
Specificity	82	73–89
Positive Predictive Value (PPV)	68	52–82
Negative Predictive Value (NPV)	92	84–97
Accuracy	84	77–90

## Data Availability

The data supporting the findings of this study are available within the article. Additional anonymized data can be provided by the corresponding author upon reasonable request.

## Abbreviations

The following abbreviations are used in this manuscript:

AVN	Avascular necrosis
CT	Computed tomography
HU	Hounsfield unit
IMN	Intramedullary nailing
NPV	Negative predictive values
ORIF	Open reduction and internal fixation
PHFs	Proximal humeral fractures
PPV	Positive predictive values
RSA	Reverse shoulder arthroplasty
VAS	Visual Analog Scale
